# 

**Published:** 1903-08-08

**Authors:** 


### NOTICE TO CORRESPONDENTS.

All MSS., Letters, Books for Review, and other matters intended for the Editor should be addressed The Editor, "The Hospital," The Hospital Buildings, 28 & 29 Southampton Street, Strand, W.C.

The Editor cannot undertake to return rejected MS., even when accompanied by stamped directed envelope.

**Notice to Advertisers and Subscribers.**

All Advertisements, Orders for Copies of The Hospital, and Business Communications should be addressed to **THE MANAGER, (Not to the Editor)**, The Hospital, 28 & 29 Southampton Street, Strand, London, W.C.

The Cost of Subscription, post free, is as follows.—Per week, 3½d.; per quarter, 3s. 9d.; per half-year, 7s. 6d.; per annum, 15s. Foreign and Colonial:—Per annum, 19s. 6d., payable, in all cases, in advance.

**NOTICE.—Vol. XXXIII. of "The Hospital" (October 1902 to March 1903) in handsome cloth binding, will shortly be on sale at the office of this Journal, price 7s. 6d. Cases for binding the Numbers contained in this Volume 1s. 6d. Index to Vol. XXXIII., 2½d., post free.**

**The monthly part for August is now ready. Price 1s., by post 1s. 3d.**

### THE STUDENT OF MEDICINE.

### APPOINTMENTS.

**T. M. Allison**, M.D., B.S.Durh., Physician to the Hospital for Diseases of Women, Newcastle-on-Tyne. **William Butler**, M.B., C.M.Glasg., D.P.H.Lond., Medical Officer of Health for Willesden. **C. Hawkins Craig**, M.B., Ch.B., House Surgeon to the Edinburgh Royal Maternity and Simpson Memorial Hospital. **M. Corner Edred**, B.Sc.Lond., M.A., M.B.Cantab., F.R.C.S.Eng., Assistant Surgeon to the Hospital for Sick Children, Great Ormond Street. **Hugh Clayton Fox**, F.R.C.S.I., Assistant Surgeon to the Metropolitan Ear, Nose, and Throat Hospital, Grafton Street, W. **Leonard West**, M.B., Ch.B., House Surgeon to the Edinburgh Royal Maternity and Simpson Memorial Hospital.

### "The Hospital" Institutional Library.

"Hospitals and Asylums of the World." 4 Vols., with a Portfolio of Plans, complete £12 12s.

"Burdett's Hospitals and Charities, 1902." 5s.

"Burdett's Official Nursing Directory, 1903." 3s. net.

"Cottage Hospitals, General, Fever, and Convalescent." 10s. 6d.

"Uniform System of Accounts for Hospitals and Public Institutions." New Edition. 4s. net.

"Hospital Expenditure: The Commissariat." 2s. 6d.

"A Legal Handbook for the Use of Hospital Authorities." 2s. 6d.

"The Cottage Hospital Case Book or Register of Patients." 10s. and 12s. 6d.

All these are published by the SCIENTIFIC PRESS, Ltd., and may be obtained through any bookseller, or direct from the publishers, 28 and 29 Southampton Street, Strand, London, W.C.

### To Become a Nurse

You should obtain

### THE NURSING PROFESSION: HOW AND WHERE TO TRAIN.

By SIR HENRY BURDETT, K.C.B.

A thoroughly up-to-date Guide to Training for this important profession, with full particulars of Training Schools in the United Kingdom and abroad.

Price **2/4** post free.

### As a Probationer

You will need

### NURSING: ITS THEORY AND PRACTICE.

By PERCY G. LEWIS, M.D., M.R.C.S., L.S.A.

Being a complete text-book of Medical, Surgical, and Monthly Nursing.

Price **3/6** post free.

### ELEMENTARY ANATOMY AND SURGERY FOR NURSES.

By WM. MCADAM ECCLES, M.S., M.B., F.R.C.S., L.R.C.P.

A valuable text-book to Nurses who are beginning the study of these subjects. The instruction is given very clearly and concisely.

Price **2/6** post free.

### ELEMENTARY PHYSIOLOGY FOR NURSES.

By C. F. MARSHALL, M.D.

This book contains all that a Nurse requires to know of this important subject.

Price **2/-** post free.

### THE NURSE'S DICTIONARY OF MEDICAL TERMS AND NURSING TREATMENT.

By HONNOR MORTEN.

An indispensable companion for every Nurse.

Price **2/-** post free; in leather, **2/8** post free.

### PRACTICAL GUIDE TO SURGICAL BANDAGING AND DRESSINGS.

By WM. JOHNSON SMITH, F.R.C.S.

A ready and complete pocket reference book for Nurses.

Price **2/-** post free.

*These books are obtainable of all Booksellers, and are published by* THE SCIENTIFIC PRESS, LIMITED, 28 & 29 Southampton Street, Strand, London, *who will send their latest Catalogue of Nursing Text-books post free to any Nurse on application.*

